# Cancer associated fibroblasts secreted exosomal miR-1290 contributes to prostate cancer cell growth and metastasis via targeting GSK3β

**DOI:** 10.1038/s41420-022-01163-6

**Published:** 2022-08-23

**Authors:** Shuo Wang, Peng Du, Yudong Cao, Jinchao Ma, Xiao Yang, Ziyi Yu, Yong Yang

**Affiliations:** Key Laboratory of Carcinogenesis and Translational Research (Ministry of Education), Urological Department, Peking University Cancer Hospital & Institute, Bejing, 100142 PR China

**Keywords:** Cancer microenvironment, Cell migration

## Abstract

Cancer-associated fibroblasts (CAFs) play crucial roles in mediating tumor growth and metastasis via transferring exosomes to neighboring cells, whereas the mechanisms by which CAFs regulate the tumorgenesis of prostate cancer (PC) remain largely unknown. In this study, CAFs and normal fibroblasts (NFs) were isolated from PC tissues and adjacent normal tissues, respectively. Exosomes (NFs-Exo and CAFs-Exo) were then isolated from the supernatant of NFs and CAFs. Next, the differentially expressed miRNAs (DEMs) between NFs-Exo and CAFs-Exo were identified using RNA-sequencing. Cell viability, migration and invasion were detected with CCK-8 and Transwell assays. Protein expression was measured with western blot. We found that CAFs-Exo remarkably enhanced PC cell migration, invasion, stemness, epithelial-mesenchymal transition (EMT) and metastasis. Significantly, miR-1290 level was upregulated in CAFs-Exo compared to NFs-Exo. In addition, CAFs could transfer exosomes to PC cells, resulting in a marked increase of miR-1290 level in cells. Moreover, exosomal miR-1290 could inhibit GSK3β/β-catenin signaling by binding with the downstream target GSK3β mRNA. Meanwhile, miR-1290 antagomir notably reversed the effects of CAFs-Exo on PC cells through activating GSK3β/β-catenin signaling. Collectively, exosomal miR-1290 from CAFs could promote PC cell growth and metastasis via inhibiting GSK3β/β-catenin signaling, suggesting that miR-1290 may serve as potential therapeutic target for the treatment of PC.

## Introduction

Prostate cancer (PC) is the most common non-cutaneous cancer diagnosed in men with an estimated new 1,600,000 cases annually [1, 2]. Although the therapies for the treatment of PC including androgen deprivation therapy (ADT) surgical prostatectomy, radiotherapy and hormone therapy improve overall survival outcomes, severe side effects remain inevitable [3–5]. Therefore, identifying novel therapeutic targets for PC may promote the development of approaches for early detection and therapies.

Increasing evidences indicated that the cellular interaction within the tumor microenvironment (TME) exhibits key roles in reprograming tumor initiation, growth and metastasis [6, 7]. CAFs are the main stromal cells in the TME that facilitate the progression of human cancers via interacting with cancer cells [8, 9]. Recently, evidences have shown that CAFs could facilitate prostate tumor growth and invasion [10–12]. However, the mechanisms by which CAFs regulate the tumorgenesis of prostate tumor remain largely unknown.

CAFs could promote tumor growth and metastasis through communicating with cancer cells within TME [13]. In addition, the crosstalk between CAFs and cancer cells are often mediated by extra-cellular signals including exosomes [14]. Exosomes are 40–130 nm nano-sized extracellular vesicles that are secreted by cells [15]. Additionally, exosomes can act as mediators for intercellular crosstalk by the delivery of biomolecules, such as proteins, microRNAs (miRNAs), mRNA, DNA) and lipids [16, 17]. MiRNAs are able to negatively mediate gene expression [18, 19]. Furthermore, miRNAs are highly enriched in exosomes, and exosomes can transfer functional miRNA molecules from CAFs to cancer cells [20–22].

In the present study, our results indicated that miR-1290 level was significantly elevated in CAFs-Exo compared to NFs-Exo. In addition, CAFs led to significant increases in PC cell migration, invasion, stemness and metastasis through transferring exosomal miR-1290. The present findings demonstrating that miR-1290 may serve as potential therapeutic target for PC treatment.

## Materials and methods

### Isolation of primary human fibroblasts

PC tissues and matched adjacent non-cancerous tissues (10 pairs) were collected from the patients with PC who underwent surgery. This study was approved by the Ethics Committee of Peking University Cancer Hospital & Institute, and each participant gave the written informed consent. According to previously report, primary human CAFs and NFs were isolated from tumor tissues and matched non-cancerous tissues respectively [23]. Next, the morphology of CAFs and NFs were captured with a light microscope.

### Cell culture and cell transfection

Human PC cell lines PC3, 22RV1 and LNCaP were obtained from American Type Culture Collection. These cell lines were authenticated by STR profiling and tested negative for mycoplasma contamination. PC cells were maintained in F-12K medium or in RPMI-1640 (Thermo Fisher Scientific) containing 10% FBS in a humidified 5% CO_2_ atmosphere at 37 °C. In addition, CAFs and NFs were maintained in DMEM/F12 medium containing 10% FBS at 37 °C with 5% CO_2_.

MiR-1290 agomir (50 nM), miR-1290 antagomir (anti-miR-1290, 100 nM) and negative control (NC) was purchased from RIBOBIO. Meanwhile, GSK3β was ligated into the pcDNA3.1 vector to obtain pcDNA3.1-GSK3β (GSK3β-OE) plasmids (GenePharma). Next, NC, miR-1290 agomir, anti-miR-1290 or GSK3β-OE plasmids were transfected into PC cells using Lipofectamine 2000.

### Immunofluorescence (IF) assay

CAFs and NFs were probed with primary antibodies against vimentin (Abcam) and α-SMA (Abcam) overnight at 4 °C. Next, cells were incubated with secondary antibody conjugated with fluorescent Alexa Fluor® 594 (Abcam) in darkness. Finally, cells were captured with a fluorescence microscope. Nuclei were stained with DAPI for 15 min.

### Exosome isolation and identification

CAFs and NFs were cultured in DMEM/F12 medium containing 10% FBS until about 80% confluent at 37 °C. Then, medium was replaced with DMEM/F12 medium without FBS. Next, exosomes were collected from the supernatant of CAFs and NFs using ultracentrifugation. Briefly, culture supernatant was centrifuged at 300 × *g* for 10 min, at 2000 x *g* for 10 min, and at 10,000 *g* for 30 min. Culture supernatant was then centrifuged at 100,00 x *g* for 70 min. The precipitates were collected and washed with sterilized PBS (ASPEN). Later on, the solution was centrifuged again at 100,00 × *g* for 70 min. The final pellet was resuspended in PBS (ASPEN). Next, exosomes were treated with 2.5% glutaraldehyde and then loaded on a carbon-coated copper grid. After that, 1% phosphotungstic acid was applied to stain the grid. Next, transmission electron microscope (TEM) was applied to examine the morphologies of the samples. Size distributions and quantification of exosomes were determined using a ZetaView nanoparticle tracking analyzer (Particle Metrix). PC3 and 22RV1 cells were treated with CAFs-Exo or NFs-Exo (50 μg/ml) for indicated times.

### Western blot assay

Proteins were electrophoresed on 10% SDS-PAGE and electroblotted onto a PVDF membrane. Next, the membrane was probed with primary antibodies including anti-TSG101 (cat. no. ab125011, 1:1000), anti-CD9 (cat. no. ab236630, 1:1000), anti-E-cadherin (cat. no. ab231303, 1:1000, anti-N-cadherin (cat. no. ab76011, 1:1000), anti-Vimentin (cat. no. ab92547, 1:1000), anti-CD133 (cat. no. ab216323, 1:1000), anti-OCT4 (cat. no. ab200834, 1:1000), anti-Glycogen synthase kinase-3 beta (GSK3β; cat. no. ab93826, 1:1000), anti-β-catenin (cat. no. ab223075, 1:1000), anti-c-Myc (cat. no. ab32072, 1:1000), anti-cyclin D1 (cat. no. ab16663, 1:1000), anti-Calnexin (cat. no. ab133615, 1:1000) and anti-GAPDH (cat. no. ab8245, 1:1000) overnight at 4 °C. The membrane was then incubated with a secondary antibody for 1 h. Finally, the blots were developed with an enhanced chemiluminescence kit. All antibodies were provided from Abcam. GAPDH was used as an internal control.

### Exosome labeling and uptake

NFs-Exo and CAFs-Exo were labeled with PKH26 membrane dye (Sigma). Next, PC3 cells were treated with above exosomes for 24 h, and then photographed by a fluorescence microscope. Phallodin was used to label F-actin of the cytoskeleton, DAPI was used to label nuclei.

### RNA-sequencing (RNA-seq)

Total RNA was extracted from CAFs-Exo or NFs-Exo with the TRIpure Total RNA Extraction Reagent (ELK Biotechnology). RNA-seq libraries were prepared from all samples using the NEBNext Ultra Directional RNA Library Prep Kit for Illumina. Next, sequencing was performed by Illumina Hiseq sequencer (Illumina). The DEMs between CAFs-Exo and NFs-Exo were screened by the limma package. DEMs were obtained with |log2 (fold change)| > 1 and adjusted *p*-value < 0.05.

To predict the possible functions of the target genes of DEMs and the potential pathways they may participate in, gene ontology (GO) and Kyoto Encyclopedia of Genes and Genomes (KEGG) enrichment analysis were conducted [24].

### Cell-counting Kit-8 (CCK-8) assay

PC3 and 22RV1 cells were plated onto 96-well plates overnight. Cells were then treated with CAFs-Exo or NFs-Exo (50 μg/ml) for 48 h at 37 °C. Later on, each well was added with CCK-8 reagent (10 μL, Beyotime), and cells underwent 2 h of incubation at 37 °C. Subsequently, the absorbance of each well was measured by a microplate reader at the wavelength of 450 nm.

### Transwell assays

PC3 and 22RV1 cells (100 µL) suspended in serum-free media were added to the 24-well Transwell inserts (Corning). The lower chamber contained the culture medium with 10% FBS. Next, cells on the bottom surface of the chamber were stained with 0.2% crystal violet at 24 h. Later on, the migrated or invaded cells were examined using a microscope. For detecting cell invasion ability, Transwell inserts were pre-coated with matrigel (BD Bioscience).

### Real-time quantitative polymerase chain reaction (RT-qPCR) assay

Total RNA was isolated by TRIpure Total RNA Extraction Reagent and transcribed using EntiLink™ 1st Strand cDNA Synthesis Kit. Later on, qPCR was conducted by the EnTurbo™ SYBR Green PCR SuperMix kit and analyzed with the StepOne™ Real-Time PCR System. The level of each miRNA was normalized against U6, using 2^–ΔΔCT^ method. U6 forward, 5’-CTCGCTTCGGCAGCACAT-3’ and reverse, 5’-AACGCTTCACGAATTTGCGT-3’; miR-135b-3p forward, 5’-CCGATGTAGGGCTAAAAGCC-3’ and reverse, 5’-CTCAACTGGTGTCGTGGAGTC-3’; miR-549a-3p forward, 5’-CCGGTGACAACTATGGATGAG-3’ and reverse, 5’-CTCAACTGGTGTCGTGGAGTC-3’; miR-1290 forward, 5’-GGCTGGATTTTTGGATCAGG-3’ and reverse, 5’-CTCAACTGGTGTCGTGGAGTC-3’; miR-224-5p forward, 5’-CAAGTCACTAGTGGTTCCGTTTAG-3’ and reverse, 5’-CTCAACTGGTGTCGTGGAGTC-3’; GSK3β forward, 5’-GCTGCACAGGAAAAACCACC-3’ and reverse, 5’-CCCCCTGGATCTCCCTCAAA-3’. All kits were provided from ELK Biotechnology.

### Sphere-forming assay

PC3 and 22RV1 cells were cultured in DMEM/F12 medium containing 4 ng/ml insulin (Sigma), 2% B27 (Thermo Fisher Scientific), 20 ng/ml EGF (Sigma), 10 ng/ml FGF (Sigma). After 14 days of incubation, cells were observed under a light microscopy.

### Luciferase reporter and TOPflash reporter assays

The fragment of 3′UTR of GSK3β containing the binding site of miR-1290 was inserted into the pGL6-miR‐based luciferase reporter vector. Next, PC3 cells were co-transfected with well-designed pGL6-miR‐based reporter plasmids, along with a miR-1290 agomir using Lipofectamine 2000 for 48 h. Subsequently, the Dual Luciferase Reporter Assay System (Beyotime) was used to measure luciferase activity in cell lysates. For the TOPflash reporter assay, PC3 cells were co-transfected with TOPflash reporter gene (TCF Reporter Plasmid; Millipore), along with anti-miR-1290. At 48 h post-transfection, the reporter activities were assayed by Dual Luciferase Reporter Assay System (Beyotime). Renilla luciferase activity was used for normalization.

### RNA pull-down assay

The biotinylated-miR-1290 probe was incubated with Dynabeads™ M-280 (Thermo Fisher Scientific) at 4 °C for 30 min. PC3 cells were collected, lysed, and sonicated. After that, the biotin-beads complex was added to the lysates and incubated at 4 °C overnight with gentle mixing by vortex. Subsequently, the RNA mix bound to the beads was eluted and extracted with TRIzol reagent (Thermo Fisher Scientific). Later on, RNA samples were reverse-transcribed into cDNA using the EntiLink™ 1st Strand cDNA Synthesis Kit (ELK Biotechnology). Next, PCR was performed using the EntiLink™ PCR Master Mix kit (ELK Biotechnology). Subsequently, PCR products were examined by 1% agarose gel electrophoresis, and then visualized by ultraviolet (UV).

### Animal models

Male BALB/c nude mice (18–22 g, 6 weeks old) were obtained from the Charles River. PC3 cells (1 × 10^7^ cells in 200 μL PBS) were injected into the left flank of each mouse subcutaneously. When the tumor reached about 200 mm^3^, mice were divided into 4 groups (*n* = 6 per group; the sample size used in animal study is usually based on the experience in previous research) randomly: control, NFs-Exo, CAFs-Exo and CAFs-Exo + anti-miR-1290. After that, NFs-Exo or CAFs-Exo (1 mg/kg) was injected into the tumor twice a week. Meanwhile, 50 nM miR-1290 agomir was injected directly into the tumor twice per week. Meanwhile, tumor volume was calculated as (length x width^2^)/2. Animals were sacrificed on day 21, and the tumors were isolated and weighted.

Regarding as liver metastasis model, nude mouse was anesthetized with 1% pentobarbital sodium. After that, the spleen of mouse was sub-capsularly injection of PC3 cells (5 × 10^6^ cells). Mice were sacrificed at day 21 post injection and the number of liver metastatic nodules in these mice was determined. This study was approved by the Ethics Committee of Peking University Cancer Hospital & Institute, and animals were maintained following the institutional guidelines. The investigator was blinded to the group allocation.

### Immunohistochemistry (IHC) assay

The tumor tissues were embedded in paraffin and cut into 4 μm sections. Later on, tissues were probed with the primary antibody against Ki67 overnight at 4 °C. Next, sections were probed with HRP conjugated anti-rabbit second antibody for 30 min. Subsequently, tissues were visualized with DAB solution and photographed under a light microscope.

### Statistical analyses

All data were repeated in triplicate. Data are expressed as the mean ± standard deviation (S.D.). Group comparisons among three or more groups were analyzed by One-way analysis of variance (ANOVA) and Tukey’s test. Differences between tumor tissues and adjacent normal tissues were analyzed using paired Student’s t-test (normal distribution). Data are reported including estimation of variation within each group. A P-value less than 0.05 was considered to have statistical significance.

## Results

### CAFs-Exo promoted PC cell migration and invasion

**Fig. 1:**
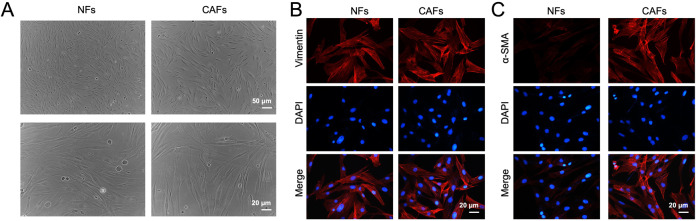
Characteristics of fibroblasts derived from patients with PC. **A** Representative morphology of CAFs and NFs isolated from patients with PC. Upper panels, scale bar = 50 μm; lower panels, scale bar = 20 μm. **B**, **C** IF staining for vimentin and α-SMA expressions of CAFs and NFs. Scale bar = 20 μm.

CAFs play key roles in tumor progression through transferring exosomes to neighboring cells [25]. To explore the role of CAFs in PC progression, NFs and CAFs were collected from patients with PC firstly. Then, cell morphologies were observed using a microscope. CAFs and NFs displayed the typical spindle-shape morphology (Fig. 1A). Additionally, CAFs and NFs were positive for vimentin, which was verified by IF assay (Fig. 1B). Meanwhile, α-SMA level was much higher in CAFs than that in NFs (Fig. 1C). Collectively, CAFs and NFs were isolated successfully.

**Fig. 2:**
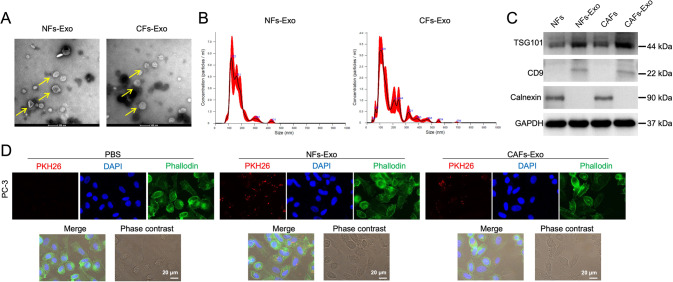
Isolation of exosomes from CAFs and NFs. **A**, **B** Identification of exosomes (CAFs-Exo and NFs-Exo) derived from CAFs and NFs by TEM (Scale bar = 100 nm) and NTA analysis. Yellow arrow points at exosomes. **C** Western blot analysis of TSG101, CD9 and Calnexin expressions in CAFs-Exo and NFs-Exo. **D** PC3 cells were treated with PKH26-labeled CAFs-Exo and PKH26-labeled NFs-Exo for 24 h, and the red exosome signal was captured by fluorescence microscopy. Scale bar = 20 μm.

Next, exosomes were isolated from the supernatant of CAFs (CAFs-Exo) and NFs (NFs-Exo), and the isolated vesicles were identified using TEM, nanoparticle tracking analysis (NTA) and western blot analysis. The results indicated that these vesicles (40–130 nm in diameter) were round and cup-shaped (Fig. 2A, B). In addition, exosomal markers TSG101 and CD9 were highly expressed, but exosomal negative marker calnexin was negative expressed in these vesicles. (Fig. 2C and Supplemental Material). These results suggested that the isolated vesicles were exosomes.

To determine if exosomes could be absorbed by PC3 cells, PKH26-labeled CAFs-Exo or PKH26-labeled NFs-Exo were added into PC3 cells. The results revealed that PKH26 dye was observed in CAFs-Exo- and NFs-Exo-treated PC3 cells, indicating that exosomes released by CAFs and NFs could be internalized by PC3 cells (Fig. 2D).

**Fig. 3:**
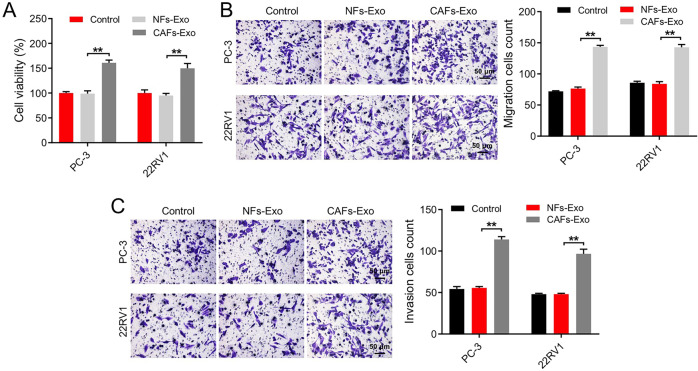
CAFs-Exo enhanced PC cell migration and invasion. **A** PC3 and 22RV1 cells were treated with CAFs-Exo or NFs-Exo (50 μg/ml) for 48 h. CCK-8 assay was used to determine cell viability. **B**, **C** PC3 and 22RV1 cells were incubated with CAFs-Exo or NFs-Exo (50 μg/ml) for 24 h. Transwell assays were used to determine cell migratory and invasive abilities. Scale bar = 50 μm. **P < 0.01. Data are presented as mean ± S.D. (*n* = 3). One-way ANOVA was used for comparing differences among three groups.

We next to explore the effects of CAFs-Exo on PC cells (PC3 and 22RV1 cells). Compared to NFs-Exo, CAFs-Exo remarkably enhanced PC cell proliferation, migration, and invasion (Fig. 3A–C). Collectively, CAFs-Exo facilitated PC cell migration and invasion.

### Identification of the DEMs between CAFs-Exo and NFs-Exo

**Fig. 4:**
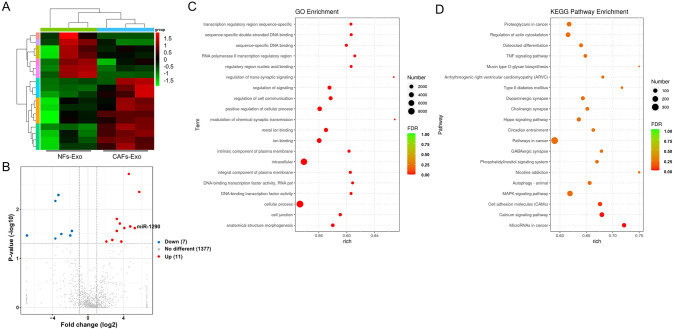
Identification of DEMs between NFs-Exo and CAFs-Exo. **A** Heatmap showing the miRNA expression profiles of NFs-Exo and CAFs-Exo. **B** Volcano plot of DEMs between CAFs-Exo and NFs-Exo. **C** Gene ontology (GO). **D** Kyoto Encyclopedia of Genes and Genomes (KEGG) analysis.

As we know, exosomes could shuttle miRNAs to neighboring cells in the TME [26]. To explore the mechanisms how CAF-Exo exerts their pro-tumor effects on PC cells, the DEMs between CAFs-Exo and NFs-Exo was investigated using RNA sequencing assay. As shown in Fig. 4A, B, 7 downregulated miRNAs and 11 upregulated miRNAs were detected in CAF-Exo compared with NFs-Exo. In addition, GO results showed that the target genes of these 18 DEMs were primarily enriched in “regulation of cell communication”, “intracellular” and “cellular process” (Fig. 4C). KEGG pathway analysis revealed that the target genes of DEMs were mainly involved in “microRNAs in cancer” pathway (Fig. 4D).

### CAFs secreted exosomal miR-1290 promoted PC cell migration, invasion, EMT, and stemness

**Fig. 5:**
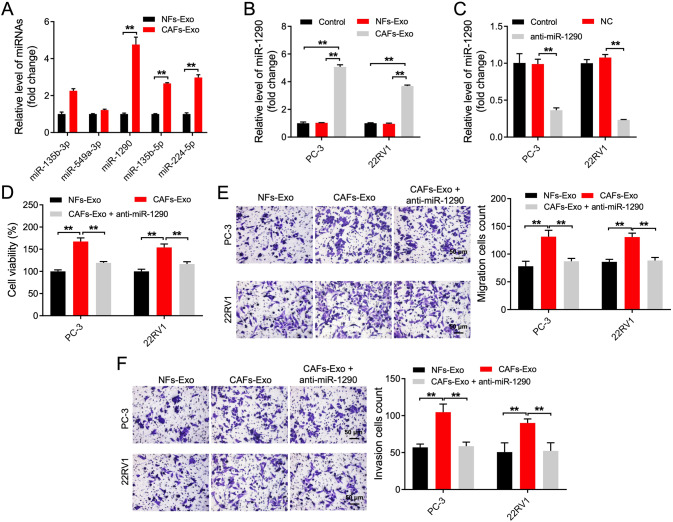
CAFs secreted exosomal miR-1290 promoted PC cell migration, invasion. **A** RT-qPCR analysis of miR-135b-3p, miR-549a-3p, miR-1290, miR-135b-5p and miR-224-5p level in CAFs-Exo and NFs-Exo. **B** RT-qPCR analysis of miR-1290 level in PC3 and 22RV1 cells incubated with indicated exosomes (50 μg/ml) for 48 h. **C** RT-qPCR analysis of miR-1290 level in PC3 and 22RV1 cells transfected with anti-miR-1290. **D** PC3 and 22RV1 cells were treated with NFs-Exo, CAFs-Exo or CAFs-Exo + anti-miR-1290 for 48 h. Cell viability was determined using CCK-8 assay. **E**, **F** PC3 and 22RV1 cells were treated with NFs-Exo, CAFs-Exo or CAFs-Exo + anti-miR-1290 for 24 h. Cell migratory and invasive abilities were measured by Transwell assays. Scale bar = 50 μm. **P < 0.01. Data are presented as mean ± S.D.; *n* = 3. Student’s *t*-test and one-way ANOVA were used for comparison among the different groups.

With the aim of exploring the DEMs, cell experiments were conducted accordingly. Among these DEMs, miR-1290, miR-135b-5p and miR-224-5p level was notably elevated in CAFs-Exo compared to NFs-Exo (Fig. 5A). In addition, we found miR-1290 level was obviously elevated in PC tissues compared to adjacent normal tissues (Supplementary Fig. 1A). Moreover, high miR-1290 level correlated with shorter overall survival rates in patients with castration-resistant PC [27]. Meanwhile, miR-1290 level was obviously elevated in CAFs compared with that in NFs (Supplementary Fig. 1B). Thus, we focused our attention on miR-1290 in the next experiments, since the level of miR-1290 in CAFs-Exo was much higher that in NFs-Exo (Fig. 5A).

**Fig. 6:**
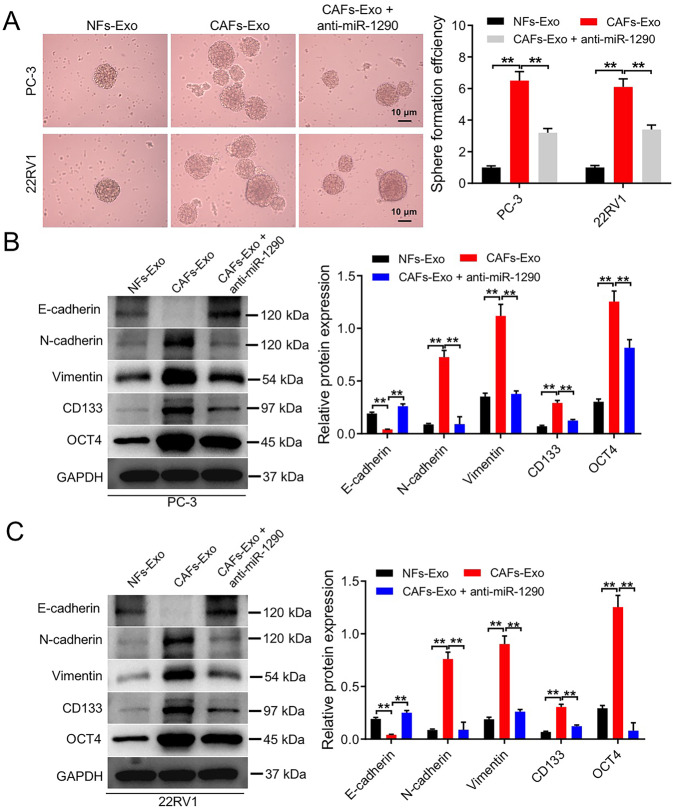
CAFs secreted exosomal miR-1290 promoted PC cell stemness and EMT. **A** PC3 and 22RV1 cells were treated with NFs-Exo, CAFs-Exo or CAFs-Exo + anti-miR-1290 for 2 weeks. The self-renewal of the PC cells was measured by a sphere-forming assay. Scale bar = 10 μm. **B**, **C** PC3 and 22RV1 cells were treated with NFs-Exo, CAFs-Exo or CAFs-Exo + anti-miR-1290 for 48 h. Western blot analysis of E-cadherin, N-cadherin, vimentin, CD133, and OCT4 expression in PC3 and 22RV1 cells. **P < 0.01. Data are presented as mean ± S.D.; *n* = 3. The difference among three groups was performed by one-way ANOVA.

Next, the result of RT-qPCR assay showed the level of miR-1290 was notably elevated in PC cells incubated with CAFs-Exo compared to cells treated with NFs-Exo (Fig. 5B). In addition, anti-miR-1290 remarkably lessened miR-1290 level in PC cells (Fig. 5C). Furthermore, CAFs-Exo significantly enhanced PC cell proliferation, migration, and invasion, whereas these phenomena were abolished by anti-miR-1290 (Fig. 5D–F). Moreover, CAFs-Exo remarkably increased the sphere-forming efficiency and notably elevated stemness markers CD133 and OCT4 expressions in PC cells; however, these phenomena were reversed by anti-miR-1290 as well (Fig. 6A–C and Supplemental Material). Meanwhile, CAFs-Exo markedly downregulated E-cadherin level and upregulated N-cadherin and vimentin level in PC cells compared to NFs-Exo, whereas the effects of CAFs-Exo were abolished by anti-miR-1290 (Fig. 6B, C and Supplemental Material). All these data illustrated that CAFs secreted exosomal miR-1290 was able to promote PC cell migration, invasion, EMT and stemness.

### GSK3β is a direct target of miR-1290 in PC cells

**Fig. 7:**
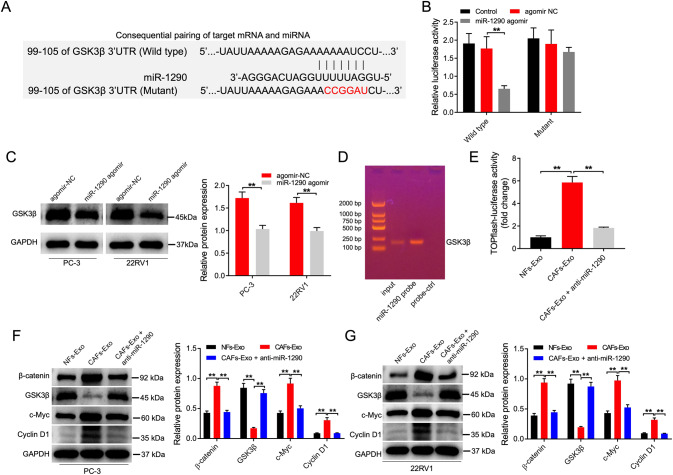
GSK3β is a direct target of miR-1290 in PC cells. **A** Sequences of miR-1290 and the potential miR-1290-binding sites at the 3’UTR of GSK3β. **B** Effect of ectopic miR-1290 expression on the luciferase activity of GSK3β 3’UTR wild type. **C** PC3 and 22RV1 cells were transfected with agomir-NC or miR-1290 agomir. Western blot analysis of GSK3β expression in PC3 and 22RV1 cells. **D** RNA pull-down assay was used to explore the interaction between miR-1290 and GSK3β mRNA. **E** PC3 cells were treated with NFs-Exo, CAFs-Exo or CAFs-Exo + anti-miR-1290 for 48 h. Dual luciferase assay was used to determine TOP reporter activity in PC3 cells. **F**, **G** PC3 and 22RV1 cells were treated with NFs-Exo, CAFs-Exo or CAFs-Exo + anti-miR-1290 for 48 h. Western blot analysis of GSK3β, β-catenin, c-Myc and cyclin D1 expression in PC3 and 22RV1 cells. **P < 0.01. Data are presented as mean ± S.D.; *n* = 3. Student’s *t*-test and one-way ANOVA were used for comparison among the different groups.

To explore the mechanism by which CAFs secreted exosomal miR-1290 promoted the growth and tumorgenesis in PC, the downstream targets of miR-1290 were predicted by TargetScan [28], miRWalk [29], and DIANA-microT databases [30], as described previously. These databases commonly indicated that GSK3β might be the direct target of miR-1290 (Fig. 7A). In addition, GSK3β has been found to exhibit a key role in the development of human cancers such as breast cancer, colorectal cancer, and PC [31–33]. RT-qPCR result showed that GSK3β level was notably downregulated in PC tissues compared to normal tissues (Supplementary Fig. 1C). Moreover, dual-luciferase reporter assay showed that the luciferase activity was lower in PC cells co-transfected with GSK3β‐WT and miR-1290 agomir (Fig. 7B). Furthermore, miR-1290 agomir markedly reduced GSK3β level in PC cells (Fig. 7C and Supplemental Material). Additionally, RNA pull-down assay results revealed that GSK3β could be pulled down by miR-1290 directly (Fig. 7D). Collectively, GSK3β is a direct binding target of miR-1290.

Next, TOPflash reporter assay was used to examine the effect of exosomal miR-1290 on the activity of the Wnt pathway in PC3 cells. The result suggested that CAFs-Exo remarkably increased transcriptional activity of TOP, while anti-miR-1290 could reverse the enhancement of the transcriptional activity of TOP caused by CAFs-Exo (Fig. 7E). Meanwhile, CAFs-Exo significantly elevated β-catenin, c-Myc and Cyclin D1 level and reduced GSK3β level in PC cells compared to NFs-Exo, whereas these changes were reversed by anti-miR-1290 (Fig. 7F, G and Supplemental Material).

Furthermore, the effects of CAFs-Exo on cellular proliferation, migration and invasion were evaluated in an androgen-dependent PC cell line (LNCaP). As shown in Supplementary Fig. 2A, GSK3β level was notably reinforced in LNCaP cells when transfected with GSK3β-OE plasmids. Meanwhile, CAFs-Exo significantly facilitated LNCaP cell proliferation, migration, and invasion (Supplementary Fig. 2B, C). Consistently, anti-miR-1290 abolished the promotion effect of CAFs-Exo on LNCaP cell proliferation. Importantly, the effects of anti-miR-1290 on cells were all abolished when GSK3β was overexpressed (Supplementary Fig. 2B, C). To sum up, CAFs secreted exosomal miR-1290 was able to promote the growth and tumorgenesis of PC cells through inhibiting GSK3β/β-catenin signaling.

### CAFs secreted exosomal miR-1290 promoted PC cell growth and metastasis in vivo through downregulation of GSK3β

**Fig. 8:**
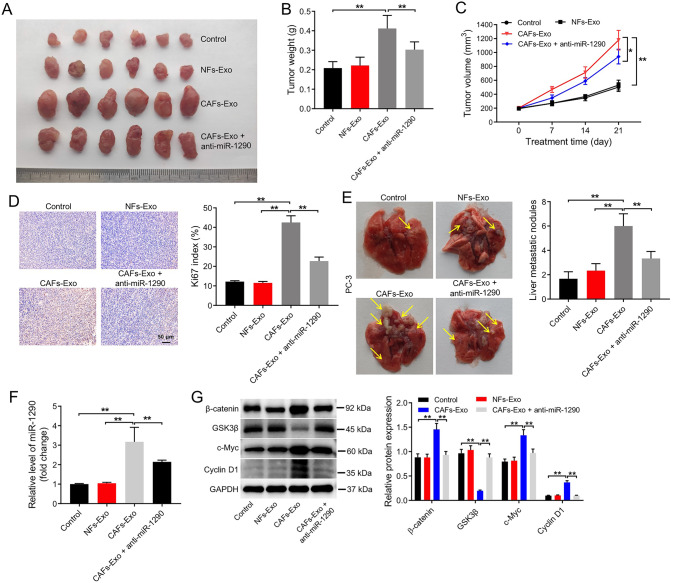
CAFs secreted exosomal miR-1290 promoted PC cell growth and metastasis in vivo through downregulation of GSK3β. **A**–**C** Tumor volume and tumor weight of xenograft tumors. **D** IHC analysis of Ki67 expression in tumor tissues. Scale bar = 50 μm. **E** The formation of metastasis nodules in liver tissues. **F** RT-qPCR analysis of miR-1290 level in tumor tissues. **G** Western blot analysis of GSK3β, β-catenin, c-Myc and cyclin D1 expression in tumor tissues. *P < 0.05; **P < 0.01. Data are presented as mean ± S.D.; *n* = 3. The significance among multiple groups was determined with one-way ANOVA.

Finally, we explored the effect of CAFs-Exo on the growth and metastasis of PC in vivo. Upon subcutaneous transplantation of PC3 cells, CAFs-Exo notably increased the tumor volume and weight compared to control or NFs-Exo group, whereas, that effects were significantly inhibited by anti-miR-1290 (Fig. 8A–C). In addition, IHC assay result revealed that the number of Ki67-positive tumor cells was much higher in CAFs-Exo-treated group than that in the control or NFs-Exo group; however, this phenomenon was reversed by anti-miR-1290 (Fig. 8D). Meanwhile, a mouse model of liver metastasis was established by subcapsular injection of the spleen method, and the data indicated that increased liver metastatic nodules were detected in CAFs-Exo group compared to control or NFs-Exo group. Similar to Ki67 stating, the promoting effect of CAFs-Exo on tumor metastasis was significantly inhibited by anti-miR-1290 (Fig. 8E).

Furthermore, CAFs-Exo remarkably increased miR-1290, β-catenin, c-Myc and Cyclin D1 level and reduced GSK3β level in tumor tissues; however, these phenomena were all reversed by anti-miR-1290 treatment (Fig. 8F, G and Supplemental Material). Taken together, CAFs secreted exosomal miR-1290 significantly promoted PC cell growth and metastasis in vivo through downregulation of GSK3β.

## Discussion

CAFs in the TME have emerged as important player in the development of human cancers, including prostate cancer [9, 34, 35]. In addition, crosstalk between CAFs and cancer cells in the TME contributes to tumor invasion and metastasis in various cancers [36–38]. Recently, exosomes have been found to emerge as critical mediators for intercellular communication between cancer cells and CAFs through transporting biomolecules including miRNAs [22, 39]. A recent study reported that breast cancer cell-derived exosomes could accelerated the activation of CAFs [40]. Donnarumma et al reported that CAFs-Exo was able to promote the EMT process and breast cancer cell stemness [41]. Hu et al. found that CAFs-Exo could enhance colorectal cancer cell metastasis through enhancing cell stemness and EMT [42]. Yang et al. showed that CAFs-derived exosomal miR-210 was able to promote the migration and invasion of non-small cell lung cancer cells [43]. In the present study, we found that CAFs-Exo notably enhanced PC cell migration and invasion. Additionally, CAFs-Exo could induce the EMT phenotypes in PC cells via upregulation of N-cadherin and vimentin protein expressions. Meanwhile, CAFs-Exo promoted PC cell stemness via upregulation of CD133 and OCT4. All these data indicated that CAFs-Exo was able to enhance PC cell migration, invasion, EMT and stemness. However, the mechanisms by which CAFs-Exo promoted cancer growth and metastasis remain largely unclear. Thus, we focus on investigating the communication between CAFs and PC cells.

Recent studies revealed that exosomes contribute to tumor progression in cancer cells through delivering miRNAs [44, 45]. Thus, RNA-sequencing assay was performed to screen DEMs between NFs-Exo and CAFs-Exo. Our results indicated that miR-1290 level was highly expressed in CAFs-Exo compared to NFs-Exo. MiR-1290 was found to be abnormally upregulated in human cancers, such as non-small cell lung cancer, oral squamous cell carcinoma and gastric cancer [46–48]. The data from Starbase showed that there are no differences regarding as miR-1290 level between PC tissues (*n* = 5) and normal tissues (*n* = 2) (Supplementary Fig. 3A). However, the sample size was too small to draw a meaningful conclusion. Our results found that miR-1290 level was obviously upregulated in PC tissues. Similar to our result, exosomal miR-1290 was found to act as a prognostic marker in castration-resistant PC, and increased miR-1290 level was related to worse overall survival of patients with castration-resistant PC [27]. However, it is still unclear why miR-1290 level is increased in PC cells. Our results indicated that CAFs could transfer exosomes to PC cells, leading to the increase of miR-1290 in PC cells. Furthermore, CAFs-Exo promoted PC cell migration, invasion, EMT and stemness, whereas these effects were abolished by anti-miR-1290. EMT and cancer stem-like cells (CSCs) have emerged as important contributors to drive metastatic dissemination of carcinomas [49–51]. These data showed that CAFs secreted exosomal miR-1290 promoted EMT and stemness, leading to the metastasis of PC. Thus, we speculated that blocking the function of CAFs-secreted exosomal miR-1290 might serve as an alternative approach for the treatment of PC.

MiRNAs play key roles in cancer progression through regulating target gene expressions [52]. MiR-1290 could promote the proliferation of colorectal cancer cells via targeting INPP4B [53]. Additionally, Yan et al. showed that miR-1290 contributed to glioma cell migration and invasion through downregulating LHX6 [54]. Meanwhile, Wu et al. found that GSK3β was a direct target of miR-1290 in A549 cells [55]. Our results consistent with the previous research showing that GSK3β is a downstream target gene of miR-1290. GSK3β is a serine/threonine kinase involved in multiple cellular processes such as cell proliferation, cell cycle and metabolic pathways [56–58]. The data from TCGA database showed that there is no difference as for GSK3β level between PC tissues and adjacent normal tissue (Supplementary Fig. 3B). However, our results showed that GSK3β level was notably reduced in PC tissues. The reason for this disparity may be due to the individual differences in patients with PC.

Importantly, GSK3β was able to contribute to the degradation of β-catenin, which related to cell proliferartion [59–61]. Meanwhile, upregulation of the Wnt/β-catenin signaling pathway could promote prostatic cancer development [62, 63]. Thus, we focus on investigating the relationships among miR-1290, GSK-3β, and β-catenin in PC cells. MiR-1246 has been found to enhance lung cell migration and invasion by activating Wnt/β-catenin signaling via inhibiting GSK-3β expression [56]. In this study, we found that CAFs-Exo obviously reduced GSK3β level and elevated β-catenin, c-Myc and Cyclin D1 level in PC cells in vitro and in vivo; whereas these changes were reversed by anti-miR-1290. Collectively, CAFs secreted exosomal miR-1290 promoted PC cell growth and metastasis via downregulating GSK3β/β-catenin signaling.

It has been shown that one miRNA can regulate numerous mRNAs [64]; thus, more studies are needed to investigate whether exosomal miR-1290 affects the progression of PC via targeting other genes.

## Conclusion

Collectively, we found that exosomal miR-1290 from CAFs could promote tumor growth and metastasis via downregulating GSK3β/β-catenin signaling, suggesting that inhibiting CAFs-derived exosomal miR-1290 may provide a new modality for the treatment of growth and metastasis in PC.

## Supplementary information

Supplementary figure 1

Supplementary figure 2

Supplementary figure 3

Supplementary figure legends

WB Original Data File

## Data Availability

The datasets used and/or analyzed during the current study are available from the corresponding author on reasonable request.
