# Supplementary figures and images for: Cancer associated fibroblasts secreted exosomal miR-1290 contributes to prostate cancer cell growth and metastasis via targeting GSK3β
Source: Cell Death Discov. 2022 Aug 23;8:371. doi: 10.1038/s41420-022-01163-6 (PMC9399109; doi:10.1038/s41420-022-01163-6)

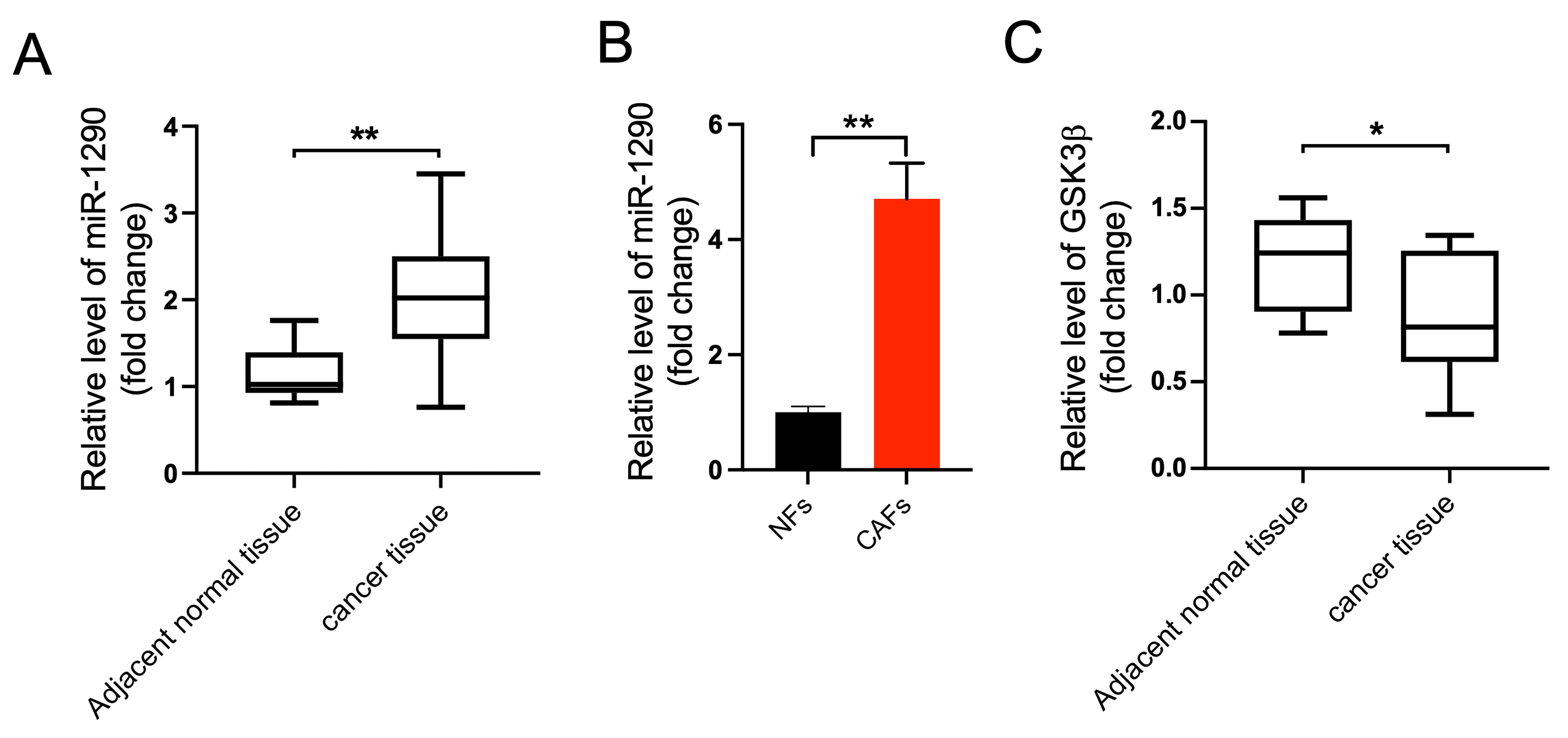

Supplement: Supplementary file 1 — Supplementary figure 1 [file 41420_2022_1163_MOESM1_ESM.jpg]

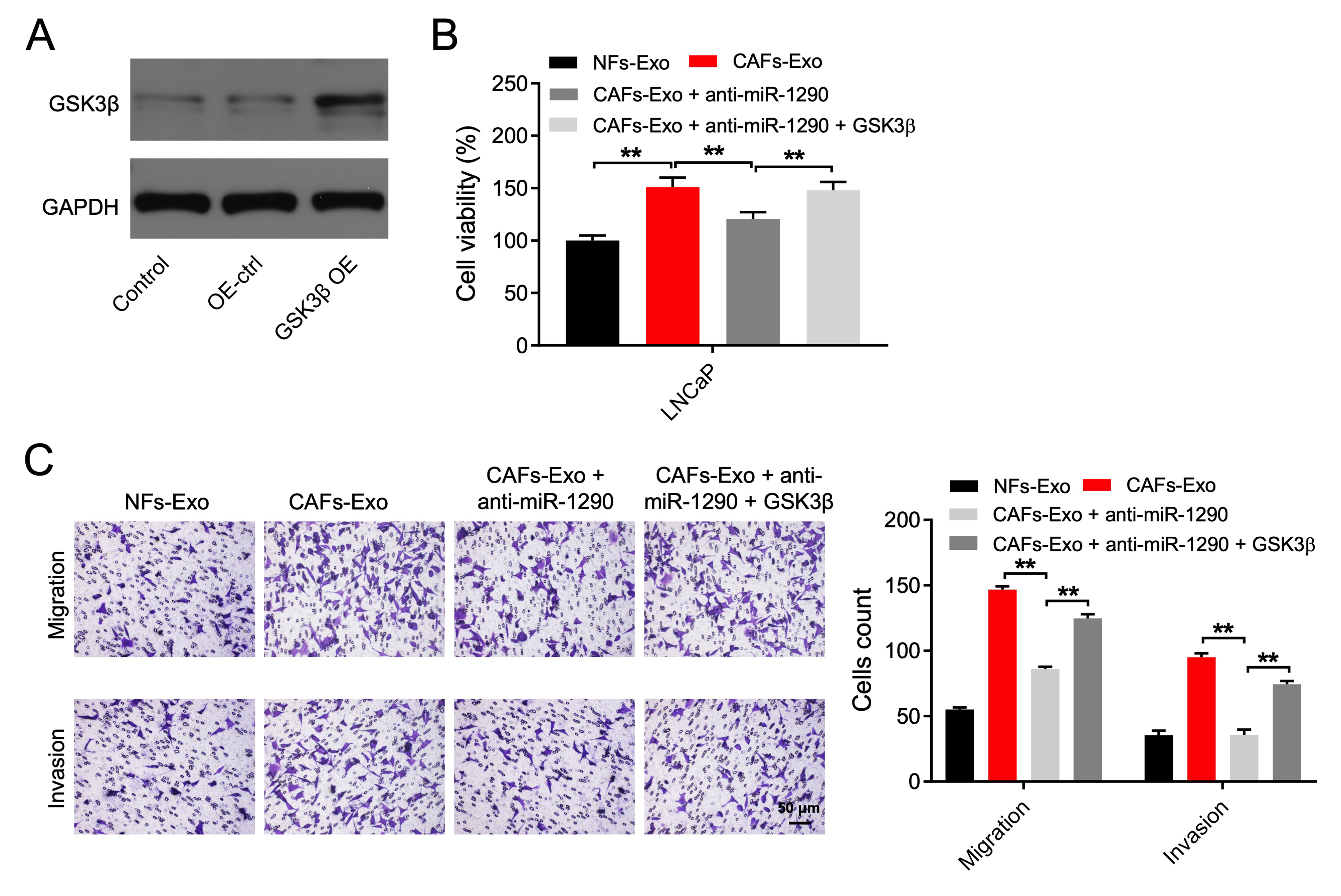

Supplement: Supplementary file 2 — Supplementary figure 2 [file 41420_2022_1163_MOESM2_ESM.jpg]

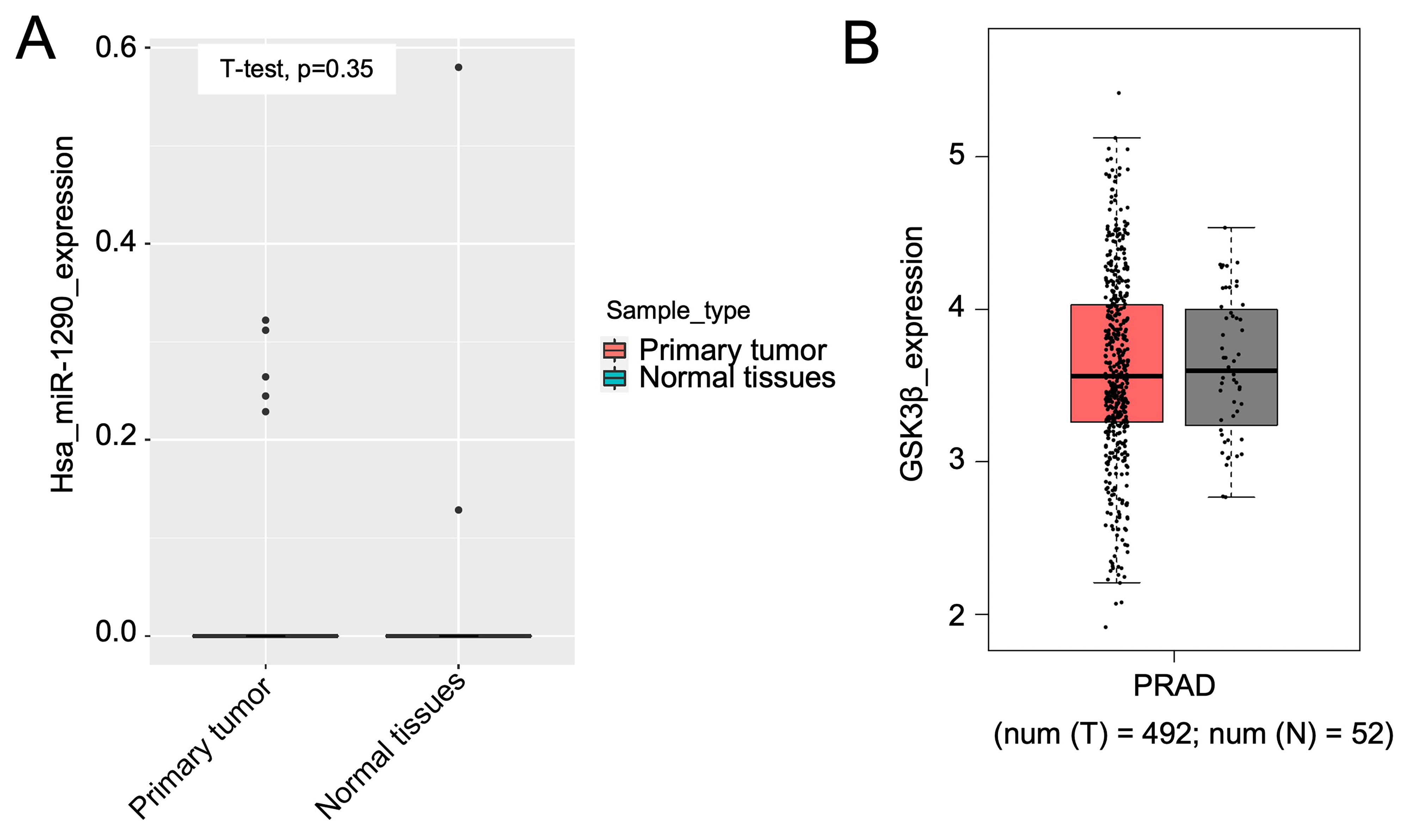

Supplement: Supplementary file 3 — Supplementary figure 3 [file 41420_2022_1163_MOESM3_ESM.jpg]
