# Supplementary material for: Cancer associated fibroblasts secreted exosomal miR-1290 contributes to prostate cancer cell growth and metastasis via targeting GSK3β
Source: Cell Death Discov. 2022 Aug 23;8:371. doi: 10.1038/s41420-022-01163-6 (PMC9399109; doi:10.1038/s41420-022-01163-6)
Supplement: Supplementary file 4 — Supplementary figure legends [file 41420_2022_1163_MOESM4_ESM.docx]

**Supplementary figure 1 The level of miR-1290 and GSK3β in PC cells. (A)** RT-qPCR analysis of miR-1290 level in PC tissues and adjacent normal tissues. **(B)** RT-qPCR analysis of miR-1290 level in CAFs and NFs. **(C)** RT-qPCR analysis of GSK3β level in PC tissues and adjacent normal tissues. *P < 0.05; **P < 0.01. Data are presented as mean ± S.D.; n = 10. Student’s t test was used for comparison between two groups.

**Supplementary figure 2 CAFs secreted exosomal miR-1290 could promote the proliferation, migration and invasion in LNCaP cells via downregulation of GSK3β. (A)** Western blot analysis of GSK3β expression in LNCaP cells transfected with GSK3β-OE plasmids. **(B)** LNCaP cells were treated with NFs-Exo, CAFs-Exo, CAFs-Exo + anti-miR-1290, or CAFs-Exo + anti-miR-1290 + GSK3β-OE. Cell viability was determined using CCK-8 assay. **(C)** Cell migratory and invasive abilities were measured by Transwell assays. **P < 0.01. Data are presented as mean ± S.D.; n = 3. The difference among four groups was performed by one-way ANOVA.

**Supplementary figure 3 The level of miR-1290 and GSK3β in PC from datasets. (A)** MiR-1290 level in PC tissues (n=5) and adjacent normal tissues (n=2) in Starbase dataset. **(B)** GSK3β level in prostate adenocarcinoma (PRAD) tissues (T) and normal tissues (N) in TCGA dataset.
