# Supplementary material for: Cancer associated fibroblasts secreted exosomal miR-1290 contributes to prostate cancer cell growth and metastasis via targeting GSK3β
Source: Cell Death Discov. 2022 Aug 23;8:371. doi: 10.1038/s41420-022-01163-6 (PMC9399109; doi:10.1038/s41420-022-01163-6)

Figure 2C TSG101

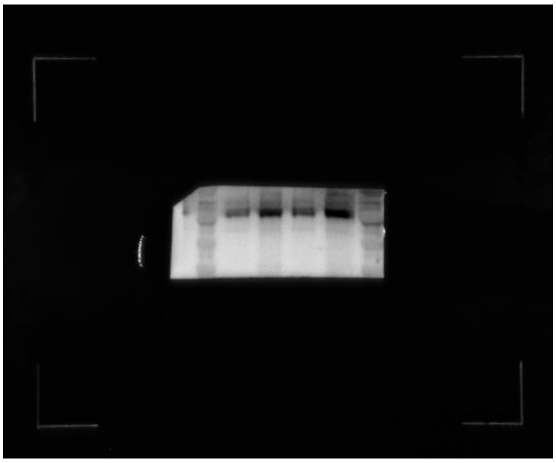

Figure 2C GAPDH

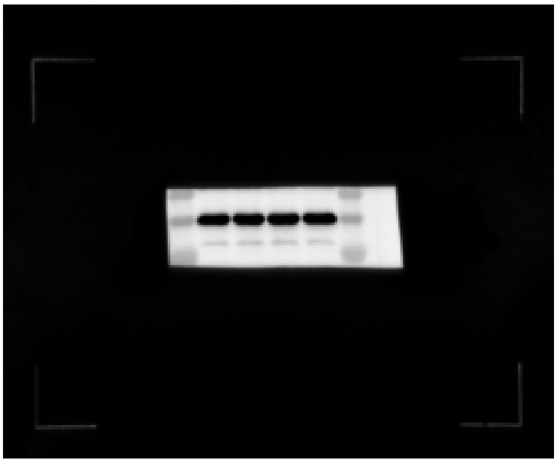

Figure 2C CD9

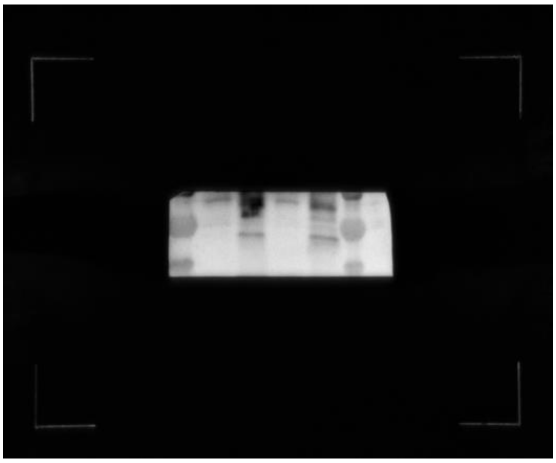

Figure 6B E-cadherin

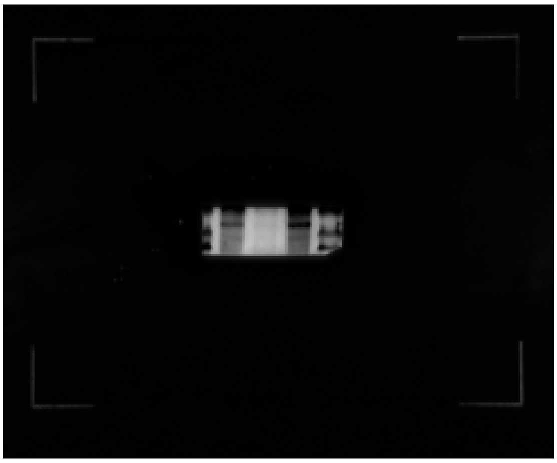

Figure 2C Calnexin

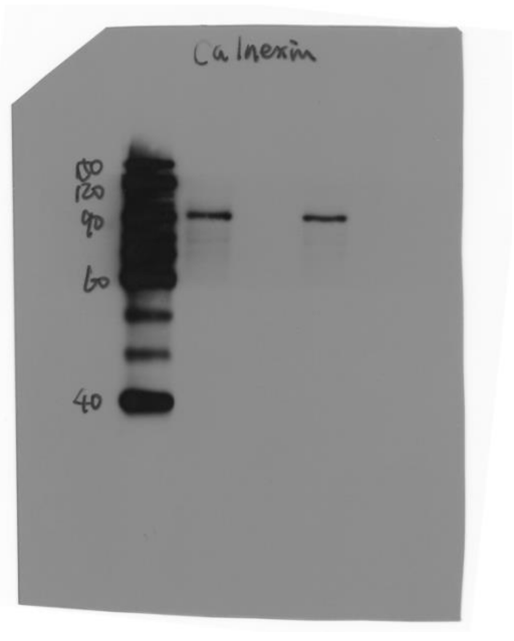

Figure 6B N-cadherin

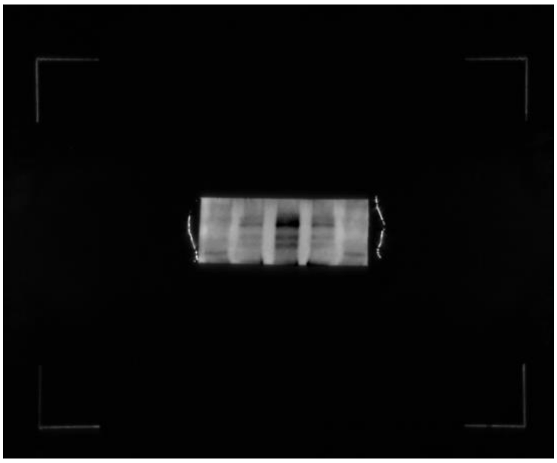

Figure 6B Vimentin

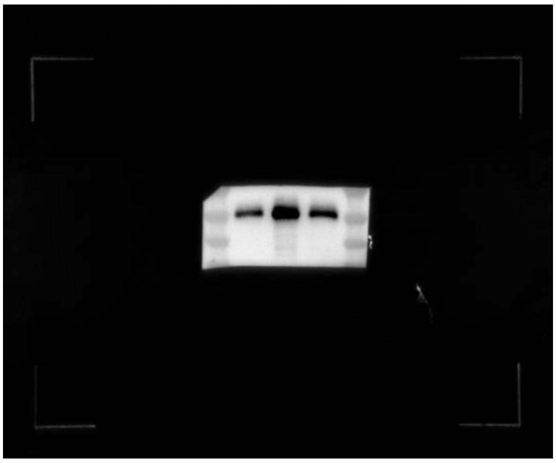

Figure 6B, C GAPDH

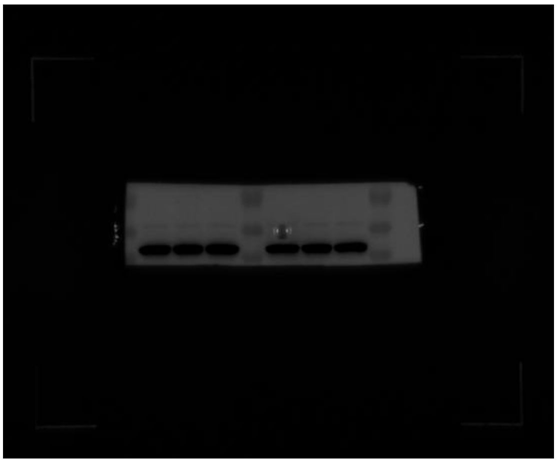

Figure 6B CD133

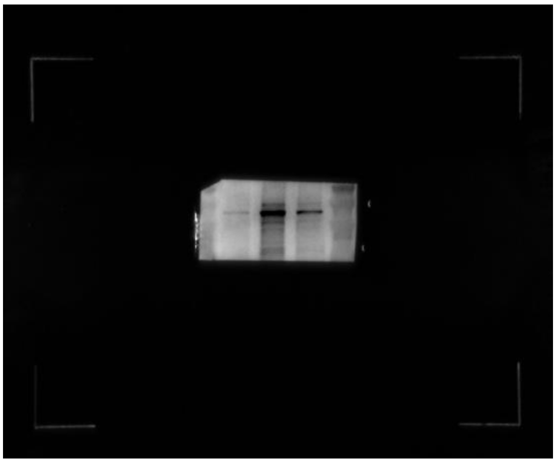

Figure 6C E-cadherin

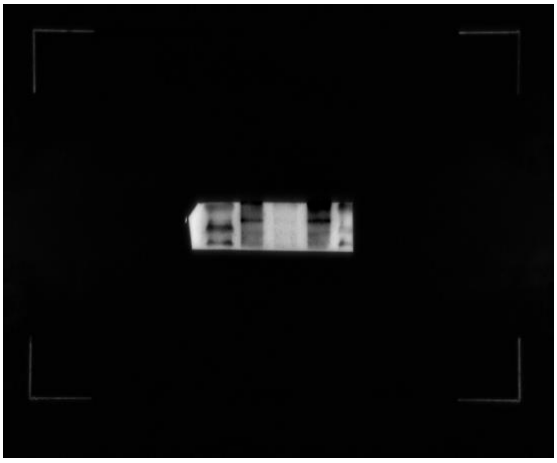

Figure 6B OCT4

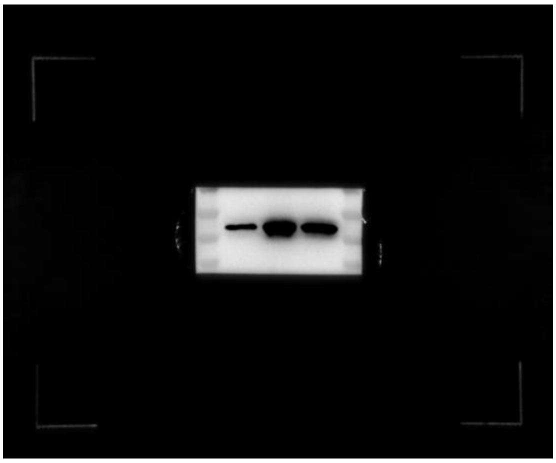

Figure 6C N-cadherin

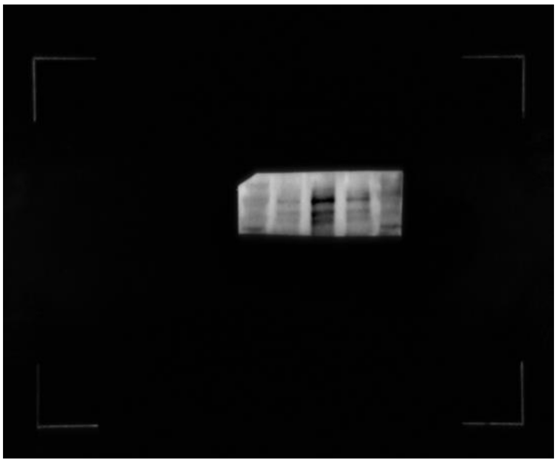

Figure 6C Vimentin

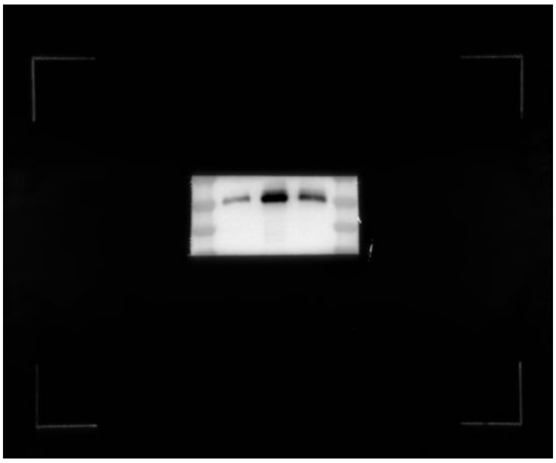

Figure 7C GSK3β (PC-3)

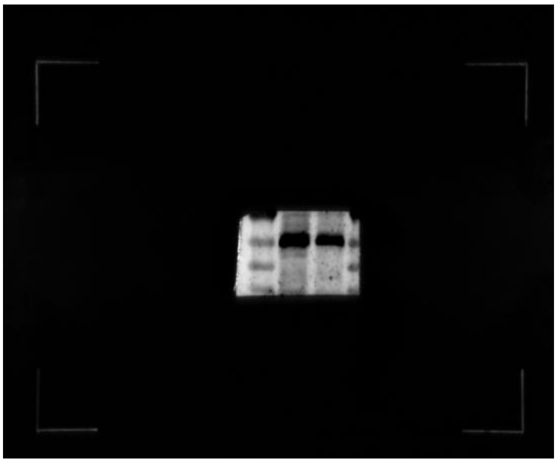

Figure 6C CD133

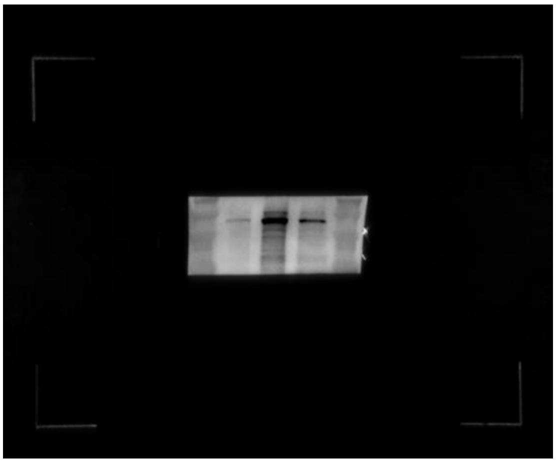

Figure 7C GAPDH (PC-3)

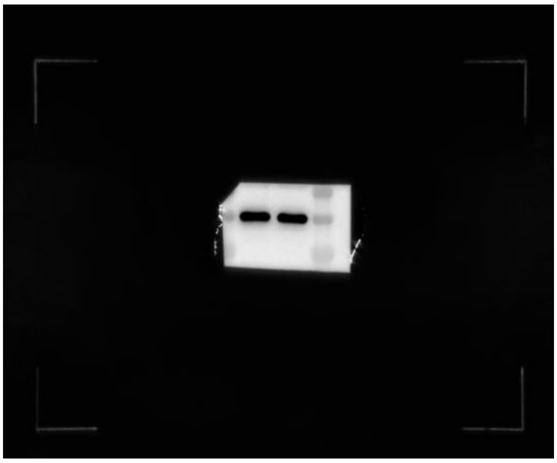

Figure 6C OCT4

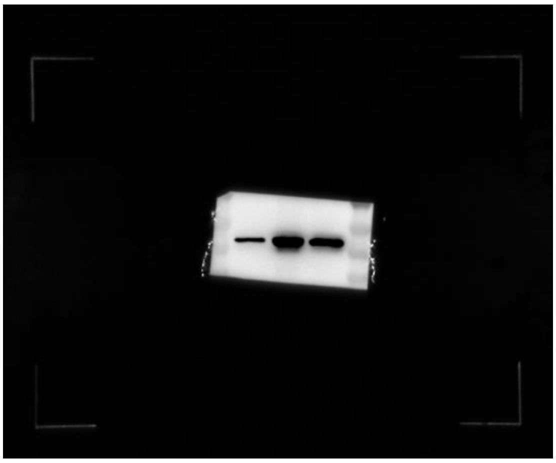

Figure 7C GSK3β (22RV1)

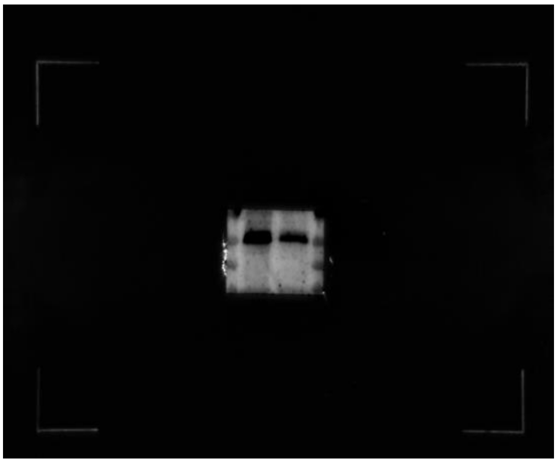

Figure 7C GAPDH (22RV1)

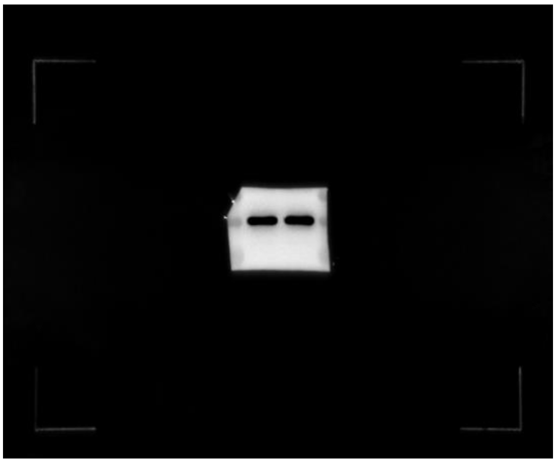

Figure 7F c-Myc

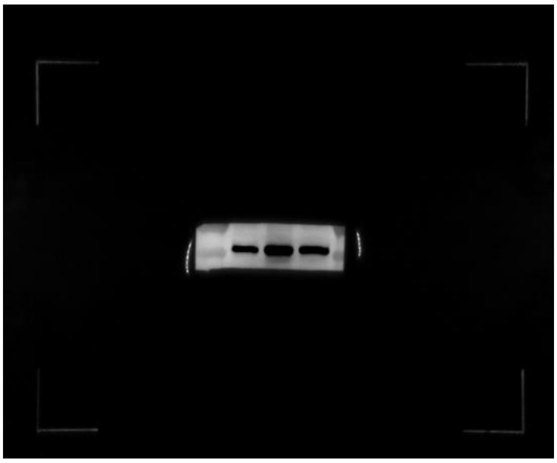

Figure 7F β-catenin

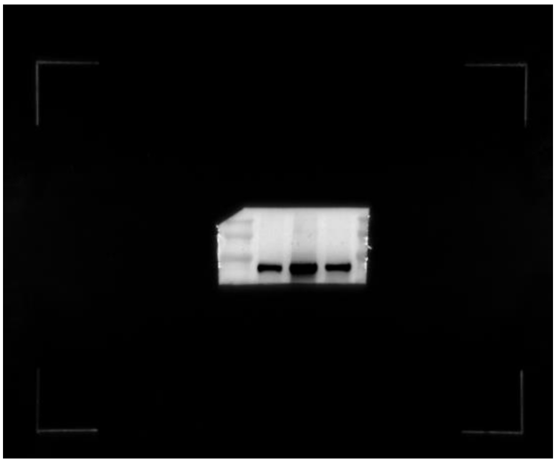

Figure 7F Cyclin D1

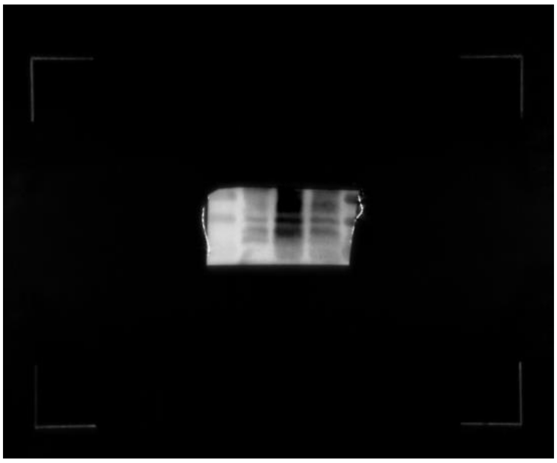

Figure 7F GSK3β

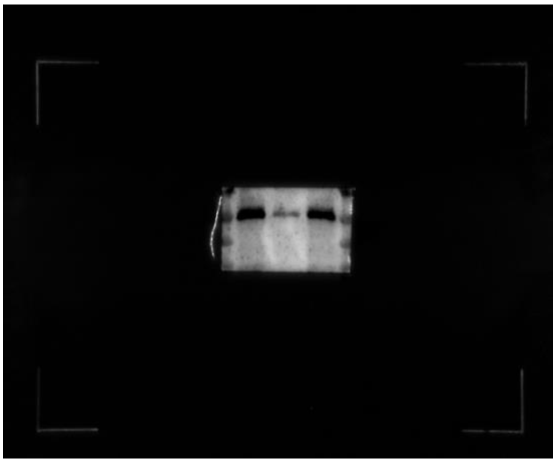

Figure 7F GAPDH

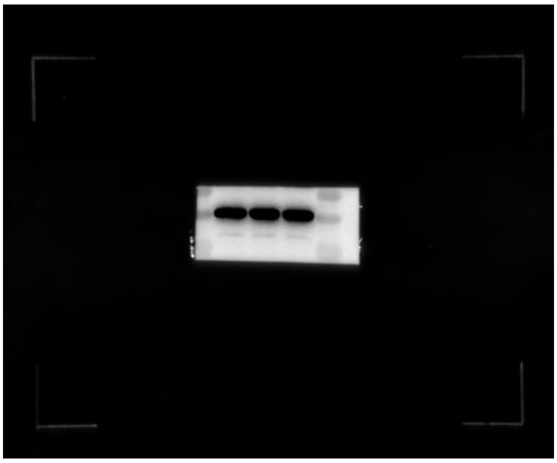

Figure 7G β-catenin

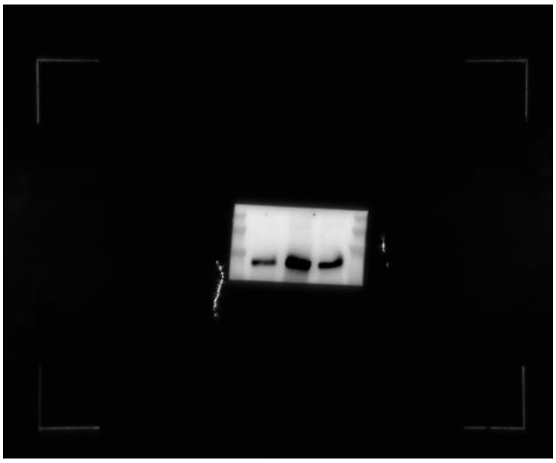

Figure 7G Cyclin D1

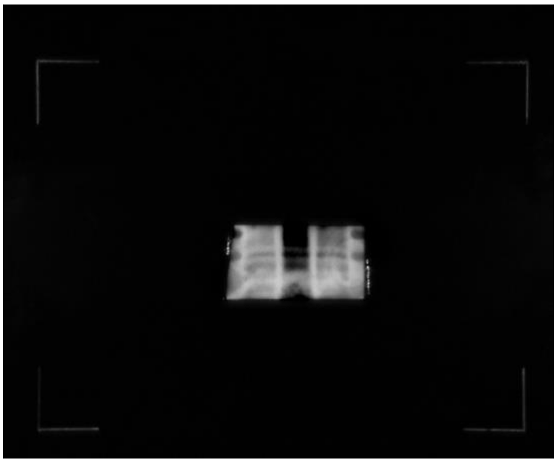

Figure 7G GSK3β

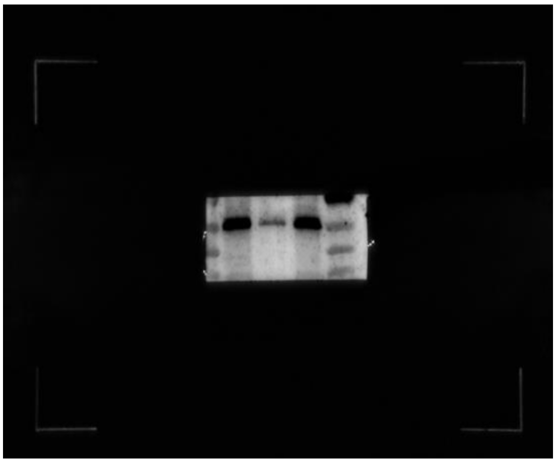

Figure 7G GAPDH

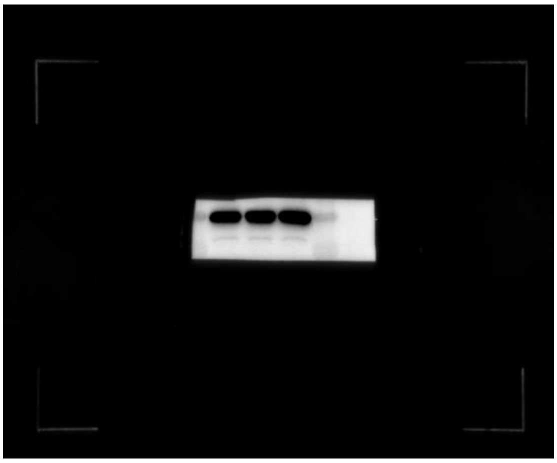

Figure 7G c-Myc

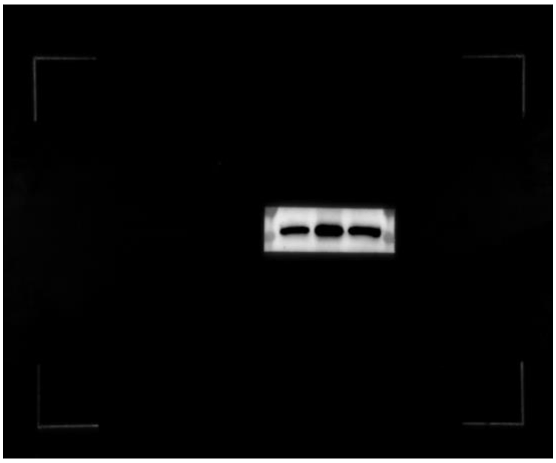

Figure 8G β-catenin

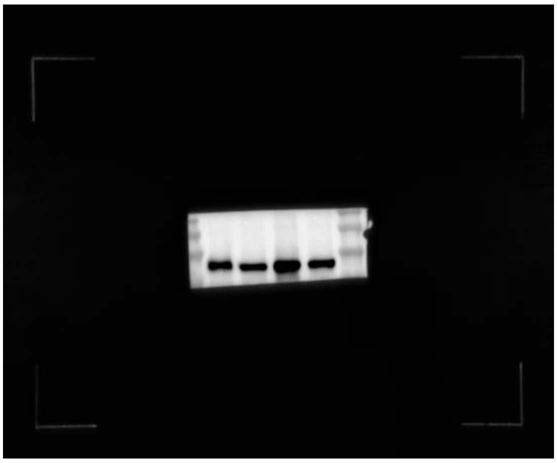

Figure 8G GSK3β

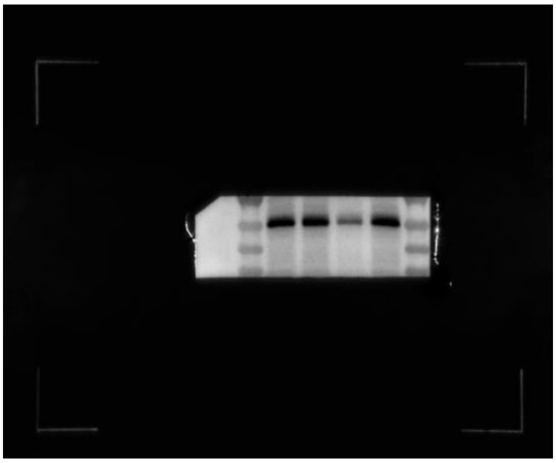

Figure 8G GAPDH

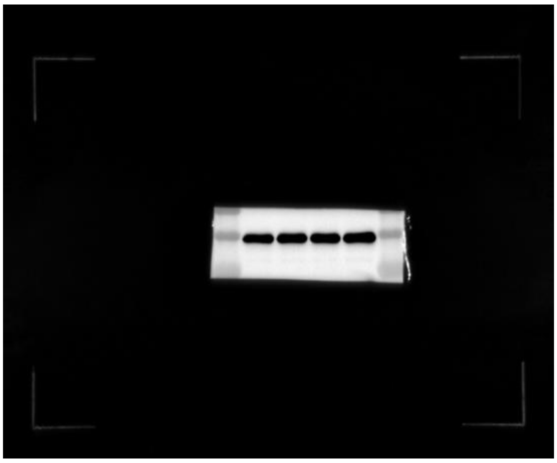

Figure 8G c-Myc

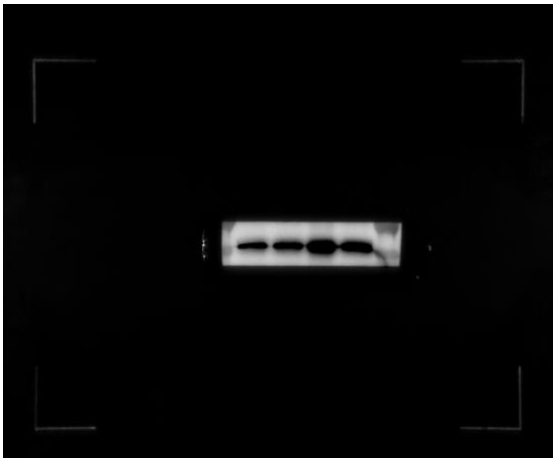

Figure 8G Cyclin D1

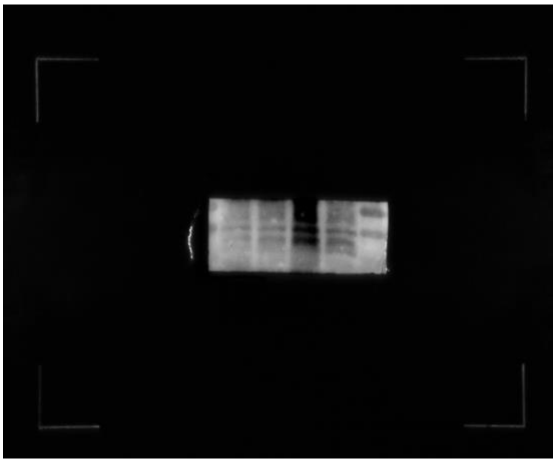

Supplement: Supplementary file 5 — WB Original Data File [file 41420_2022_1163_MOESM5_ESM.pdf]
